# A graphene microelectrode array based microfluidic device for *in situ* continuous monitoring of biofilms†

**DOI:** 10.1039/d3na00482a

**Published:** 2023-08-11

**Authors:** Jin Song, Ashaq Ali, Yaohong Ma, Yiwei Li

**Affiliations:** a Biology Institute, Qilu University of Technology (Shandong Academy of Sciences) Jinan 250103 China; b Shandong Provincial Key Laboratory of Biosensors Jinan 250103 China; c Center of Excellence in Science & Applied Technologies (CESAT) Islamabad 75000 Pakistan

## Abstract

*In situ* continuous monitoring of bacterial biofilms has been a challenging job so far, but it is fundamental to the screening of novel anti-biofilm reagents. In this work, a microfluidic system utilizing a graphene-modified microelectrode array sensor was proposed to realize the dynamic state of bacterial biofilm monitoring by electrochemical impedance. The results illustrated that the observation window period of the biofilm state is significantly prolonged due to the increment of bacterial cell load on the sensing interface, thereby greatly improving the sensing signal quality. Simulation of anti-biofilm drug screening demonstrated that the performance of this method manifestly exceeded that of its endpoint counterparts.

## Abstract 2

*In situ* continuous monitoring of bacterial biofilms has been a challenging job so far, but it is fundamental to the screening of novel anti-biofilm reagents.

## Graphical abstract

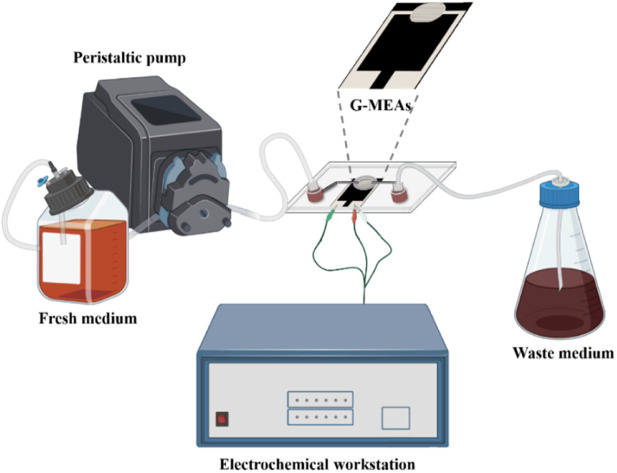

## 1. Introduction

Biofilms are communities of microorganisms that form on and adhere to surfaces and are an important survival strategy for microorganisms. Biofilms are widely found in natural, industrial and medical environments. Most of them are capable of causing contaminations,^1–4^ infections,^5–7^ and bio-corrosions.^8,9^ According to the National Institutes of Health, more than 80% of bacterial infectious diseases in the medical field are caused by biofilms.^10,11^ Biofilms are extremely difficult to eradicate due to the protective effect of bacterial extracellular polymeric substances (EPSs),^12–14^ and the existence of EPSs can greatly increase the resistance of microorganisms to biological, mechanical, physical, and chemical injuries,^3,15^ thus bringing great difficulties in treating such diseases. In addition, infections caused by biofilms impose a heavy economic burden on society. More than $8 billion is spent annually in the United States only on the treatment of oral biofilm-related diseases.^16^

There are several monitoring techniques for biofilm growth and characterization, including crystal violet staining,^17^ confocal laser scanning microscopy (CLSM),^18^ minimal biofilm eradication concentration assay,^19^ biofilm ring test,^20^ and the use of the Lubbock system and Calgary device.^21,22^ These methods enable high-throughput screening of various anti-biofilm reagents, but they are generally endpoint diagnostic tools that require destructive removal of the biofilm from the growth substrate. Therefore, dynamic bioinformatics of bacterial biofilms cannot be effectively exploited. Furthermore, most of these techniques use static culture mode with limited nutrients, due to which biofilms cannot exhibit the characteristics typically observed in natural infections. Therefore, it is particularly important to develop tools for *in situ* continuous monitoring of biofilms under near real-world conditions.

Electrochemical impedance spectrometry (EIS) is widely used in biofilm detection due to its advantages of being fast, sensitive, label-free, cost-effective and easy to miniaturize.^23–25^ The shape and material of the electrode are the predominant factors affecting the detection of bacterial biofilms by electrochemical impedance analysis. Recently, EIS technology using microelectrodes has shown many advantages in biofilm detection, especially in *in situ* and continuous monitoring uses.^26,27^ Graphene, a typical 2D material, is known to exhibit excellent electrical conductivity, high mechanical strength and biocompatibility.^28^ The combination of microelectrodes and graphene is promising for the development of new biofilm sensors. Previous studies have shown that graphene can be generated *in situ* at carbon-based electrode interfaces by electrochemical methods.^29–31^ Based on this, we have successfully constructed an electrochemically *in situ* generated graphene-modified microelectrode array (G-MEA) strategy, which is more effective in detecting the evolution of biofilms compared with conventional methods.^31^ However, dynamic information on the growth and evolution of bacterial biofilms is still unavailable.

In recent years, there has been rapid development of microfluidic technology, which enables precise control and high reproducibility of the microenvironment, making it possible for the small volume lab-on-a-chip analysis.^32^ More importantly, this technology can provide actual growth conditions for studying bacterial biofilms due to the continuous supply of nutrients, making it preferable to support the dynamic monitoring of bacterial biofilms.

Herein, we construct a microfluidic analysis system using G-MEA chips. *Streptococcus mutans* (*S. mutans*), a common oral pathogen, was used as a biofilm forming model bacterium.^33^ The growth and destruction of the biofilm were monitored *in situ* in real time using EIS assays. Furthermore, the destructive efficiencies of three representative compounds on the biofilm were accurately quantified to exemplify the merit of this methodology. Compared with destructive endpoint determination methods such as CLSM and crystal violet staining, the system was found to be able to easily achieve continuous *in situ* monitoring of the biofilm, which is expected to be applied in routine clinical analysis and anti-biofilm drug screening scenarios.

## 2. Experiments

All materials, reagents and instruments used in this work are described in detail in the ESI.†

### 2.1 G-MEA chip fabrication

(1) Spin-coating: 5 mL SU-8 3050 was spin coated on silicon wafers with an initial speed of 500 rpm and then accelerated to 2000 rpm and kept for 60 s; then the wafer was baked for 10 min at 95 °C; (2) UV exposure: the baked photoresist was exposed to UV light for 80 s in hard mode to obtain a pattern and then baked for 10 min at 95 °C; (3) developing: the sample was developed in the SU-8 developer for 7 min and then baked for 10 min at 120 °C; (4) pyrolysis: the carbon MEAs were pyrolyzed in a two-step pyrolysis process in a quartz-tube furnace (Fig. S1†). Next, they were subjected to ultrasound for 2 min, and a single carbon MEA was obtained after cutting. The MEAs consist of 35 microelectrodes with a diameter of 100 μm and an interelectrode distance of 60 μm on a silicon substrate; (5) electrochemical treatment: the MEAs after pyrolysis were placed in a 0.5 M PBS solution for electrochemical treatment. A voltage of 2.0 V was applied between the electrodes for 20 min and then negatively polarized from 0 V to −1.5 V for 10 min, respectively, with a scanning rate of 100 mV s^−1^. Electrodes were sterlized with UV irradiation light for 40 min before use.

### 2.2 Microfluidic device design and fabrication

**Fig. 1:**
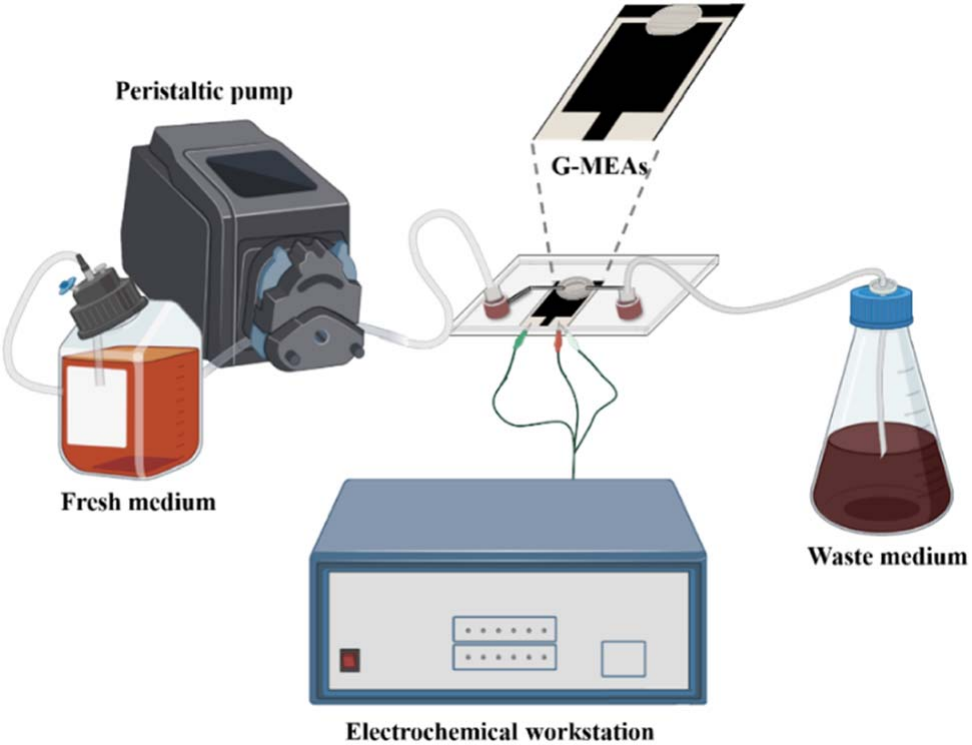
A graphical illustration of a microfluidic system for *in situ* continuous monitoring of biofilms diagram, created with Biorender (http://www.biorender.com).

The microfluidic system was assembled as shown in Fig. 1 and S2.† Briefly, the G-MEAs were placed in the center of the fixture, and their working interval was bonded to the elastic membrane covering the bottom of the chip to form a closed micro-reaction tank (7.0 mm in diameter and 0.5 mm in height), which was then fixed by tightening the four screws of the fixture. A peristaltic pump and a waste pool were connected to the Luer interface on both sides of the chip as the inlet and outlet, respectively. Prior to each experiment, the system was sterilized by pumping 75% (vol/vol) ethanol through the whole setup.

### 2.3 Biofilm formation

Single colonies of *S. mutans* were picked from the agar plate, inoculated in brain heart infusion (BHI) medium and incubated at 37 °C in an anaerobic environment with 90% N_2_, 5% H_2_ and 5% CO_2_ until the OD600 value was 0.8–1.0. Cultures were inoculated in fresh BHI medium at 1% (vol/vol) inoculum for biofilm production experiments.

### 2.4 Biofilm morphology and biomass analysis

CLSM was used to characterize the morphology of the biofilm. The steps are as follows: the fluorescent staining solution from the LIVE/DEAD BacLight Bacterial Viability Kit was added to the BHI medium inoculated with bacteria to a final concentration of 1.4 μM and 8.3 μM of Syto 9 and propidium iodide (PI) in wells. Operating away from light, the well plate was covered with foil and placed in an anaerobic incubator at 37 °C before imaging. Syto 9 was excited at 488 nm and detected at 540 nm. PI was excited at 561 nm and detected at 600 nm.^34^ In addition, metalloscopy can also be used to observe G-MEAs before and after biofilm growth.

Crystal violet staining was used to determine the total biomass adhered to the G-MEAs.^35^ Briefly, electrodes with a grown biofilm were carefully removed from the broth and washed twice with PBS to remove the loosely bound material and then air-dried at room temperature. The electrodes were stained with 30 μL, 0.1% (wt/vol) crystal violet for 15 min and washed with distilled water. Finally, those of the biofilm were destained with 3.0 mL, 33% (vol/vol) acetic acid, and the absorbance of the supernatants was measured at 590 nm on a Cytation 3 Imaging Reader. The control contained only the broth. Each test had 3 replicates.

### 2.5 *In situ* continuous monitoring of the biofilm and antibiofilm tests

The micro-reaction tank, containing bacterial suspensions, was initially cultured for 3 h. The peristaltic pump was then turned on to continuously pump fresh BHI medium at a flow rate of 100 μL min^−1^. One end of G-MEAs is used as the working electrode, and the other end is used as the counter electrode and the reference electrode. EIS measurements were performed in a Faraday cage with a ±5 mV amplitude, and a frequency range of 0.1 Hz to 100 kHz was adopted. The measurements were performed at the open circuit potential, and the bare electrode was used as a control. The other groups of micro-reaction tanks were not connected to the microfluidic system for complete static culture. Three repeats were performed. The experimental results were recorded as Δ*Z*(*Z*_Biofilm+BHI_ − *Z*_BHI_), the impedance modulus difference between the experimental group and the control group at 10.0 kHz.

Biofilm destruction experiments were performed using cetylpyridinium chloride (CPC), chlorhexidine digluconate (CHD) and cetyltrimethylammonium bromide (CTAB), which are commonly used disinfectants for biofilms.^36–38^ Preformed biofilms were exposed to the different solutions for 2 h at a flow rate of 100 μL min^−1^ at room temperature. The electrode without a biofilm was used as a control. The experimental results were recorded as Δ*Z*′(*Z*_Biofilm+Drug+BHI_ − *Z*_BHI+Drug_), the impedance modulus difference between the experimental group and the control group at 10.0 kHz. Three repeats were performed.

Subsequently, the destructive effect of the drugs on the biofilm was compared and noted as (Δ*Z* − Δ*Z*′)/Δ*Z*.

## 3. Results & discussion

### 3.1 Characterization of the G-MEAs

**Fig. 2:**
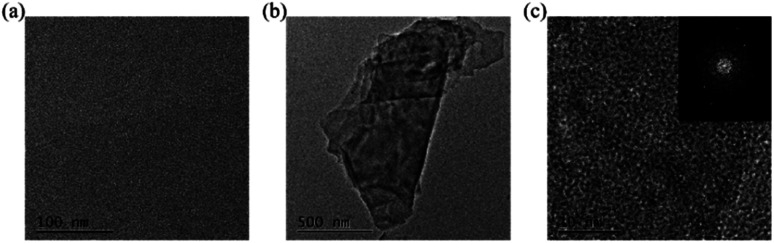
TEM characterization of the (a) post-pyrolysis MEAs and (b) G-MEAs, and (c) HRTEM of G-MEAs. The illustrations depicted in (c) show selected area diffraction (SAED) patterns.

Foremost, ultrasonication was performed on the post-pyrolysis MEAs and G-MEAs, followed by TEM characterization. Compared with post-pyrolysis MEAs (Fig. 2a), the fragments were found at the G-MEA interface (Fig. 2b). The HRTEM images from the ultrasonic exfoliated products G-MEAs (Fig. 2c) exhibit an ordered graphitic lattice. As shown in the insets of Fig. 2c, the selected area diffraction (SAED) pattern obtained from the corresponding sample exhibits multiple hexagonal sharp spots, indicating several layers of graphene sheets with ordered high crystallinity. This demonstrates that the sample we obtained by electrochemical treatments was mainly composed of several layered graphene structures, which is consistent with the results of our previous work.^31^

Secondly, the morphology of the electrode was characterized by scanning electron microscopy (SEM) as shown in Fig. S3.† As expected, the SU-8 3050 original shapes shrink after pyrolysis,^39,40^ and the post-pyrolysis MEAs have a similar structure to glass-like carbon.^41^ The structure at the G-MEA interface did not change significantly after electrochemical treatment.

Subsequently, the variations in the graphitic content in different electrochemical treatment processes were identified using Raman spectra. In Fig. S4,† all spectra obtained showed the typical D-band and G-band characteristics of carbon materials. The D peak at 1350 cm^−1^ originates from the activation of the A_1g_ mode in sp^2^ C atoms and is associated with defects and disorder in the graphite lattice,^42^ where D stands for “disordered”. The E_2g_ vibration mode at 1590 cm^−1^ leads to a G peak, which is associated with the bond stretching of sp^2^ hybridized C atoms existing in the aromatic ring and the olefinic chains, where G stands for “graphite”. The ratio of intensities *I*_D_/*I*_G_ is a well-accepted index associated with the content of defects. An increase in this ratio value indicates an increase in disorder. After oxidation, the ratio increased, indicating that defects formed during the oxidation process. After reduction, the ratio weakened back indicating the restoration of the sp^2^ bond structures and removal of the defect sites.

Furthermore, XPS was utilized to obtain detailed information about the elemental and structural composition of the structures obtained after electrochemical treatments. As can be seen from the results of XPS, the manifest changes between post-pyrolysis MEAs and G-MEAs focus on the C1s and O1s bands (Fig. S5a and b†). O1s spectra (Fig. S5b†) reveal an increase in oxygen abundance in the oxidation process and a decrease in the subsequent reduction process. As shown in Fig. S5c,† the C1 peak was deconvoluted into four peaks: sp^3^ C–C peaks at 285.1 eV, sp^2^ C–C peaks at 284.4 eV, C–O peaks at 286.4 eV and C=O peaks at 288.5 eV. The deconvoluted C1s spectra of the post-pyrolysis MEAs showed a weak sp^3^ C–C peak, the decreased intensity of the sp^2^ C–C peak and the increased sp^3^ C–C peak after oxidation, and we observed a prominent increase in carbon bonded oxygen groups. The C–O/C=O peaks increase, indicating the introduction of oxygen-containing groups (Fig. S5d†). After reduction, sp^3^ C–C peaks decreased slightly and sp^2^ C–C peaks increased slightly, implying the incomplete removal of defect/edge plane-like sites. C–O/C=O peaks declined, indicating that oxygen containing groups were partially removed (Fig. S5e†), which is also consistent with the conclusion in Fig. S5b.†

### 3.2 Biofilm morphology analysis

Previous studies found that *S. mutans* acquired a mature and stable biofilm after 12 h of *in vitro* incubation.^30,31^ Therefore, *S. mutans* was selected here for morphological characterization of its biofilm after 12 h of incubation.

**Fig. 3:**
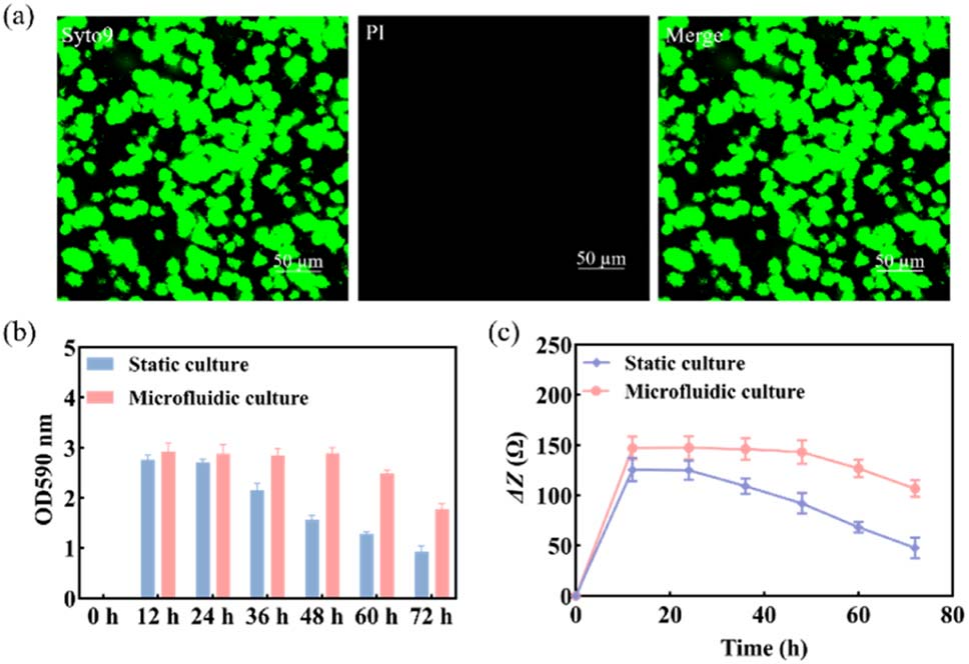
(a) The morphology of the biofilm was characterized by CLSM after 12 h of culture; (b) biomass of the *S. mutans* biofilm and (c) Δ*Z* values at different times under static and microfluidic culture conditions (*n* = 3).

The use of Syto 9 with PI can effectively distinguish between live and dead bacteria in biofilms and is therefore widely used to analyze bacterial viability. Syto 9 is a DNA-embedded dye that penetrates all bacterial membranes and stains cells green, while PI can only penetrate cells with damaged membranes, replacing Syto 9, which inhibits the emission of Syto 9 due to its stronger affinity for DNA and thus produces red fluorescent cells. Thus, the combination of the two can well distinguish bacterial cells with intact cytoplasmic membranes (green fluorescence) from those with damaged cytoplasmic membranes (red fluorescence). As shown in Fig. 3a, *S. mutans* was more likely to form microcolonies after 12 h of culture. Only Syto 9 stained the biofilm green, and the color after merging was also detected as green, so it can be considered that a uniform and stable biofilm was formed, which is also consistent with the formation time of the biofilm obtained by crystal violet staining as shown in Fig. S6.† Similarly, homogeneous and dense biofilms were obtained from the G-MEAs in the microfluidic system under metalloscopy (Fig. S7†). This demonstrates that the constructed microfluidic system is able to support the normal growth, maturation, and stability of the studied biofilms.

### 3.3 Biomass analysis of the biofilm

As shown in Fig. 3b, the biomass of the biofilm reached a maximum in 12–24 h and decreased after 24 h under static culture conditions, while under microfluidic culture conditions, the biomass reached the maximum at 12–48 h and gradually decreased after 48 h, indicating that the decline rate of biofilms was significantly slower. Therefore, microfluidic culture conditions prolonged the stabilization period of the *S. mutans* biofilm and increased its biomass. This is mainly due to the continuous input of a fresh medium to the microfluidic system micro-reaction tank and the output of metabolic waste liquid, which provides a more favorable survival environment for the biofilm. Under the microfluidic culture conditions, the growth and development model of the biofilm was ideal and the stable period was significantly extended. The stable observation window period of the biofilm increased by at least 300% compared with that of the static culture. Thus, a longer window period can be provided for biofilm state variable experiments.

### 3.4 Impedance monitoring of biofilm formation

Subsequently, we used EIS to continuously monitor the growth of the *S. mutans* biofilm under static and microfluidic culture conditions for 72 h. As can be seen from Fig. 3c, the Δ*Z* value of the biofilm of the static culture was stable after 12 h, *i.e.*, the biofilm reached maturity at this time; the Δ*Z* value decreased significantly after 24 h, which was consistent with the results of the crystal violet staining. This indicated that the ideal study time to study the destruction of the *S. mutans* biofilm was 12–24 h under static culture conditions. In contrast, for the microfluidic culture, the Δ*Z* value reached stability after 24 h and showed a decreasing trend after 48 h. The optimal study time for biofilm destruction was 12–48 h. Therefore, compared with the static culture, the biofilms cultured in the microfluidic system have a longer growth cycle, maturation, and stabilization period. These results further support the conclusion that microfluidic culture conditions can prolong the maturation cycle of the *S. mutans* biofilm.

### 3.5 Impedance monitoring of biofilm destruction by antibiofilm drugs

**Fig. 4:**
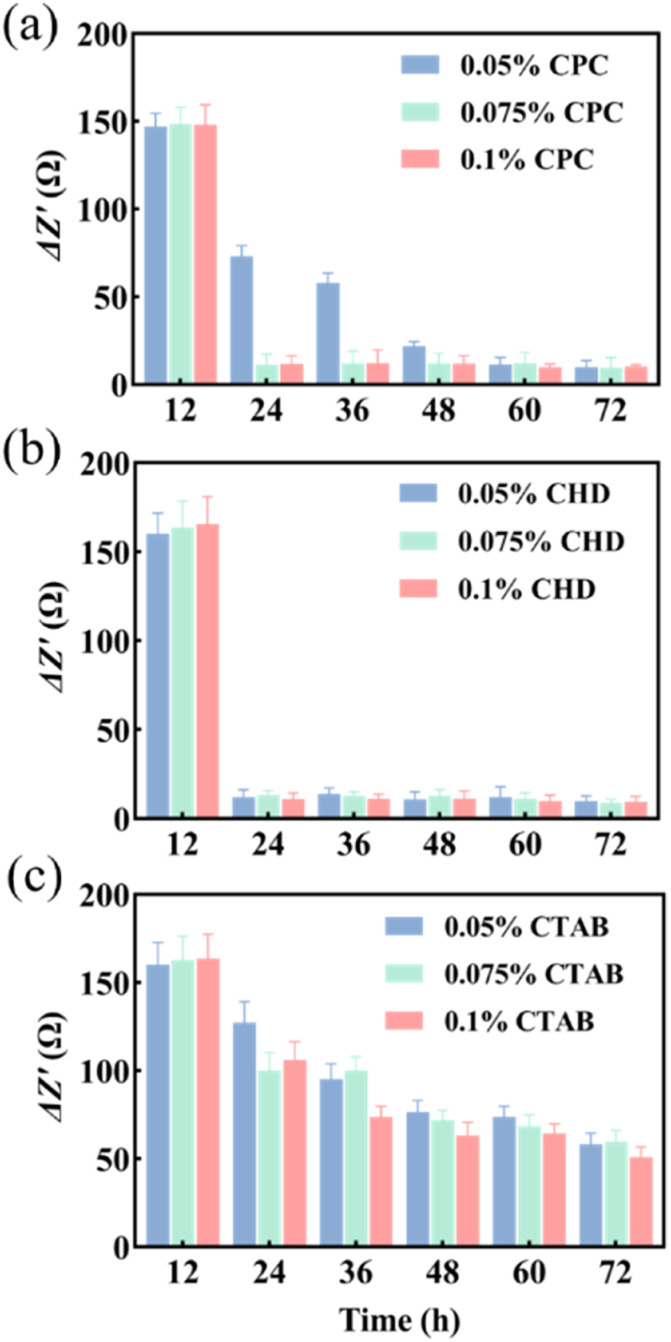
Δ*Z*′ values of different drugs. (a) CPC; (b) CHD; (c) CTAB (*n* = 3).

Three anti-biofilm drugs, CPC, CHD and CTAB, were selected to detect and compare their destruction effects on the biofilm in a microfluidic system by using EIS techniques. According to the results shown in Fig. 3b and c, 12 h was selected as the starting time for the study of biofilm destruction under microfluidic culture conditions. The results showed that CPC (Fig. 4a) and CHD (Fig. 4b) both had obvious destructive effects on the *S. mutans* biofilm and 0.05% (wt/vol) of CHD cleared most of the biofilm and achieved 92.6% efficiency at 24 h (Table 1). The effect of CTAB (Fig. 4c) was mediocre, and the biofilm destruction efficiency of three concentrations of CTAB only reached 22.5–39.1% after treatment for 12 h. This is consistent with previous reports in which the drug effects of the compounds were compared.^43,44^ The above results show that microfluidics combined with EIS can realize the continuous monitoring of biofilm destruction by anti-biofilm drugs and can accurately distinguish the different effects of different drugs.

**Table 1:** Comparison of the destruction effect of three drugs on the biofilm at different concentrations after 12 h of treatment

Concentration (wt/vol)	CPC	CHD	CTAB
0.05%	55.5%	92.6%	22.5%
0.075%	93.1%	92.0%	39.1%
0.1%	92.8%	93.4%	35.5%

## 4. Conclusions

In summary, a microfluidic analysis system for culturing biofilms was combined with G-MEAs. Compared with crystal violet staining and CLSM, the EIS based system can substantiate continuous biofilm monitoring and screening of anti-biofilm drugs. In comparison to pure G-MEA methodology, the microfluidic system requires no movement of the G-MEAs each time for impedance sensing, so it closely resembles the natural conditions of biofilm development without artificial variables. This system is an ideal tool for developing an automated and efficient anti-biofilm drug screening platform.

## Author contributions

Jin Song: conceptualization, data curation, investigation, methodology, validation, writing – original draft. Ashaq Ali: methodology, validation. Yaohong Ma: resources, software. Yiwei Li: funding acquisition, conceptualization, supervision, resources, investigation, validation, writing – review & editing.

## Conflicts of interest

There are no conflicts to declare.

## Supplementary Material

NA-005-D3NA00482A-s001
