# Supplementary material for: A graphene microelectrode array based microfluidic device for in situ continuous monitoring of biofilms
Source: Nanoscale Adv. 2023 Aug 11;5(18):4681–6. doi: 10.1039/d3na00482a (PMC10496883; doi:10.1039/d3na00482a)
Supplement: NA-005-D3NA00482A-s001 [file NA-005-D3NA00482A-s001.pdf]

Electronic Supplementary Material (ESI) for Nanoscale Advances.
This journal is © The Royal Society of Chemistry 2023

# Electronic Supplementary Information (ESI)

## A graphene microelectrode array based microfluidic device for in situ continuous monitoring of biofilms

Jin Song,[a,b] Ashaq Ali,[c] Yaohong Ma[a,b] and Yiwei Li*[a,b]

[a] *Biology Institute, Qilu University of Technology (Shandong Academy of Sciences), Jinan 250103, China*
[b] *Shandong Provincial Key Laboratory of Biosensors, Jinan 250103, China*
[c] *Center of Excellence in Science & Applied Technologies (CESAT), Islamabad, 75000, Pakistan*
*Corresponding author

## 1. Reagents and instruments

Electrochemical measurements were performed at ambient temperature with a CHI760E electrochemical workstation (Shanghai Chenhua CO., China) inside of a Faraday cage. An anaerobic incubator (Shanghai Longyue Instrument Co., China) was used to culture the bacteria at 37°C (with 90% $N_2$, 5% $H_2$ and 5% $CO_2$). Scanning electron microscopy (SEM) was conducted by a Hitachi SU8010 SEM (Japan). The morphology of the electrodes was observed by metalloscopy (MX6R, China). An X-ray photoelectron spectroscopy (XPS, ESCALAB 250Xi, USA) analysis was conducted for detailed information on the elemental and structural composition of the electrodes with AlKα radiation as the X-ray source for excitation. All of the materials were examined with Raman spectra (inVia Qontor, Renishaw plc, UK) using a 532 nm Ar laser source for subsequent analysis using DigitalMicrograph software. The absorbance of the supernatants was measured to determine the total biomass at 590 nm using Cytation 3 Imaging Reader (Biotek, USA). An FEI Tecnai G2 (USA) was used to perform transmission electron microscopy (TEM) and high-resolution transmission electron microscopy (HRTEM). The TEM samples were prepared via ultrasonication (KQ-500VDV, Kunshan Ultrasonic Instruments Co., Ltd., 40% power). Confocal scanning laser microscopy (CLSM, Nikon A1R+ Inverted Microscope with 60×/1.4 NA oil-immersion objective lens) was also conducted. 155411-Lab-Tek chambered coverglass with 8 wells (Thermo Fisher Scientific, USA) were purchased and used. Ultraviolet (UV) exposure was performed using an SUSS MA-6 (Germany). Chemical vapor deposition system (KJ-T1200R, Zhengzhou Kejia Electric Furnace Co. Ltd) was also used.

A typical oral pathogenic bacterium *Streptococcus mutans* (*S. mutans*, ATC C 25175) was purchased from Guangdong Culture Collection Center. A brain h

eart infusion (BHI, Oxoid, UK) was used for bacterial culture. A LIVE/DEAD BacLight Bacterial Viability Kit (Molecular Probes, cat. no. L-7012, Invitrogen, USA) was purchased and used. Cetylpyridinium chloride (≥ 98%) and Chlorhexidi ne digluconate (≥ 98%) were purchased from Sigma-Aldrich, USA. Cetyltrimeth ylammonium bromide (99%) was purchased from Amresco, USA. SU-8 3050 d eveloper and SU-8 photoresist were purchased from Microchem Corp. (USA). A silicon wafer (4 inches, Czochralski polished, n-type, 500±15 μm thickness, 1-1 0 Ω/cm resistance, Ferrotec Shanghai Semiconductor Wafer Co., Ltd.) was used as the substrate. Deionized water (resistivity ≥18 MΩ/cm) was used for all exp eriments. All chemicals were commercially available and were purchased from global suppliers at analytical grade purity.

## 2. Supplementary Results

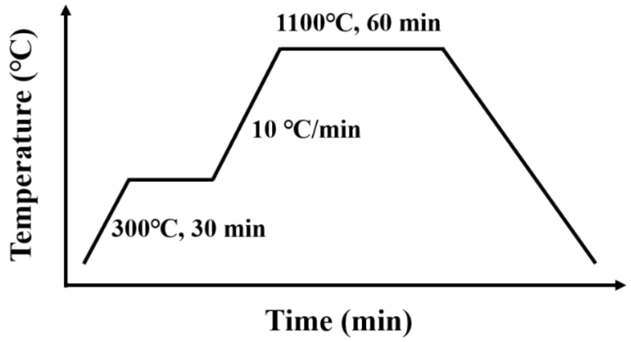


Fig. S1. schematic of a typical two-step pyrolysis process

As shown in Fig. S2, the samples were heated in an Ar atmosphere at 300 °C for approximately 30 min first and then heated in an Ar atmosphere (2000 sccm) to 1100 °C at a heating rate of 10 °C/min. All at once, the Ar gas was shut off, and 5% $H_2$ and 95% Ar were introduced (2000 sccm) for 1 h. The heater was then turned off and the samples was cooled to room temperature in an Ar atmosphere at a heating rate of 5 °C/min.

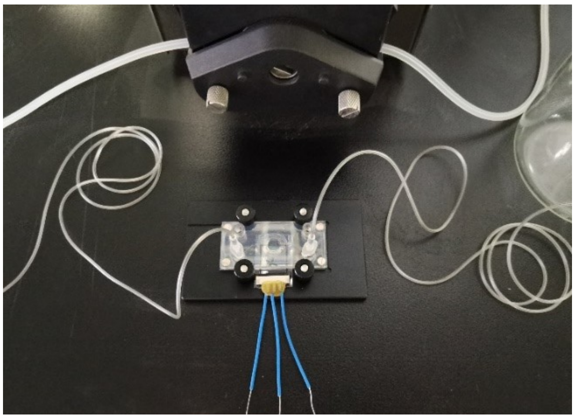

Fig. S2 Microfluidic system picture.

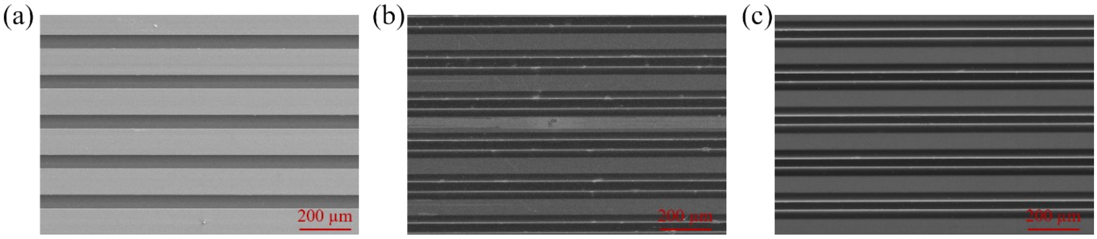


Fig. S3 (a) SEM images of the MEAs, (b) Post-pyrolysis MEAs, and (c) G-MEAs derived from SU-8 3050.

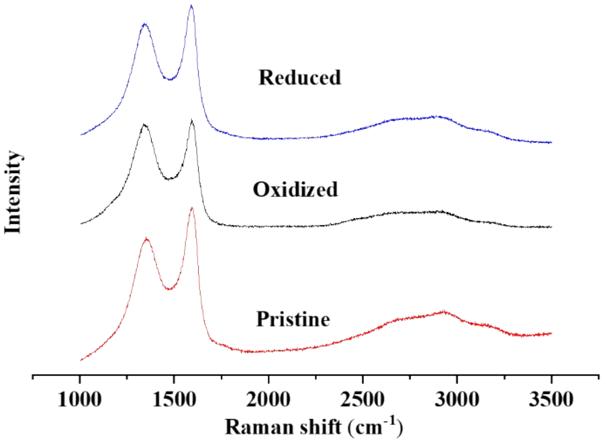


Fig. S4 Raman spectra of electrode patterns at 1100 °C pyrolysis temperatures and electrochemical treatments.

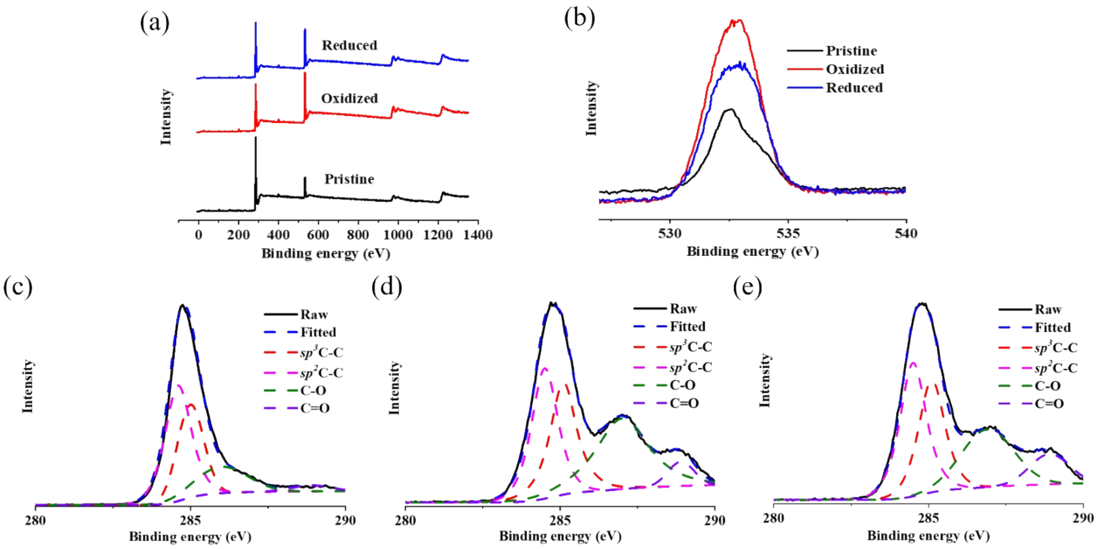


Fig. S5 (a) XPS survey scan, (b) XPS O1s spectra. XPS high-resolution C1s spectra of (c) 1100 °C pyrolysis MEAs, (d) 1100 °C-oxidized MEAs and (e) 1100 °C-reduced MEAs.

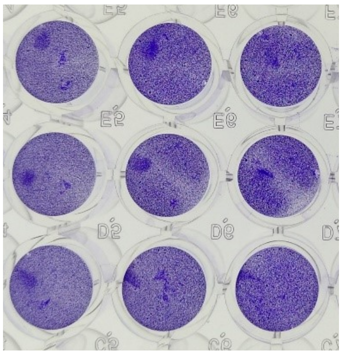

Fig. S6 The morphology of biofilm was characterized by crystal violet staining after 12 h culture.

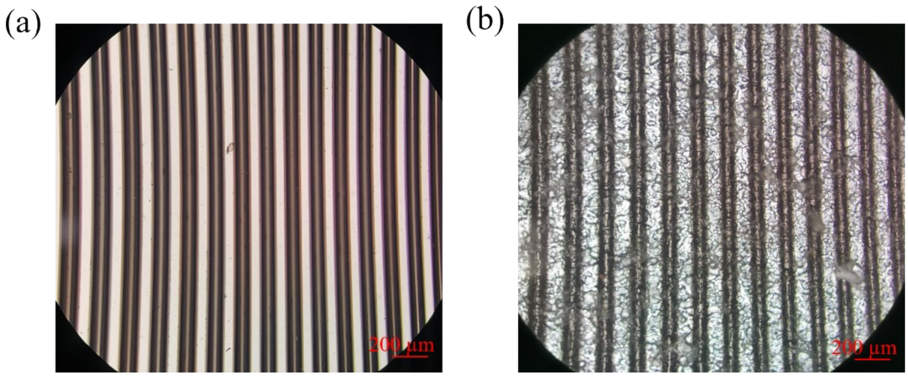


Fig. S7 The morphology of biofilm was characterized by Metalloscopy before (a) and after (b) 12 h culture.
